# Meet the Editorial Board Member

**DOI:** 10.2174/1570159X2112230822153119

**Published:** 2023-09-25

**Authors:** David J. Calkins

**Affiliations:** 1 Department of Ophthalmology and Visual Sciences, Vanderbilt University Medical Center, Nashville, TN, USA

Dr. Calkins holds a doctorate (Ph.D.) in Neuroscience from the University of Pennsylvania, Philadelpha. He is currently Professor of Ophthalmology & Visual Sciences and Pharmacology in the Vanderbilt University School of Medicine and Director of the Vanderbilt Vision Research Center. He is also Vice Chair and Director for Research of the Vanderbilt Eye Institute. He has published more over 70 primary research articles, reviews and chapters and serves on the editorial board for several journals.
